# Following the children of depressed parents from childhood to adult life: A focus on mood and anxiety disorders

**DOI:** 10.1002/jcv2.12182

**Published:** 2023-06-18

**Authors:** Victoria Powell, Jessica Lennon, Rhys Bevan Jones, Alice Stephens, Bryony Weavers, David Osborn, Judith Allardyce, Robert Potter, Ajay Thapar, Stephan Collishaw, Anita Thapar, Jon Heron, Frances Rice

**Affiliations:** ^1^ Wolfson Centre for Young People's Mental Health Cardiff University Wales UK; ^2^ Centre for Neuropsychiatric Genetics and Genomics Cardiff University Wales UK; ^3^ Cwm Taf Morgannwg University Health Board Health Board Wales UK; ^4^ Division of Psychiatry Faculty of Brain Sciences UCL UK; ^5^ Centre for Academic Mental Health Population Health Sciences Bristol Medical School Bristol University Bristol UK

**Keywords:** anxiety, childhood, depression, early adulthood, mood disorder, offspring, parent

## Abstract

**Background:**

Parental depression increases risk for anxiety and depression in offspring. The transition from adolescence to adulthood is a common risk period for onset of such disorders. However, relatively few studies have considered development of these disorders from childhood to adulthood including multiple assessments during this transition period.

**Method:**

Offspring of depressed parents aged 9–17 years at baseline were followed prospectively for 13 years (*n* = 337). Average length of follow‐up was 16 months between the first and second waves, 13 months between the second and third, and 8 years between the third and fourth. Current (3‐month) psychopathology was assessed at each wave using diagnostic interviews. We derived estimates of 3‐month prevalence, age at first diagnosis, course and comorbidity of disorders. Social functioning in adult life was assessed at the final wave and we assessed how prior and current disorder impacted adult functioning.

**Results:**

A quarter of young people met criteria for a mood disorder and a third for anxiety disorder at least once. Mood and anxiety disorder prevalence increased from 4.5% and 15.8% respectively in childhood (9–11 years) to 22.3% and 20.9% respectively by age 23–28. Increased prevalence across the transition from adolescence to adulthood was particularly marked in males, while prevalence increased earlier in adolescence in females. Age at first diagnosis varied widely (mood disorder mean = 16.5 years (range 9–26); anxiety disorder mean = 14.5 years (range 9–28)). Over half (52%) reported functional impairment in early adulthood, 31% harmful alcohol use, and 10% self‐harm or a suicide attempt. Both previous and current mood or anxiety disorder were associated with functional impairment in early adulthood.

**Conclusions:**

There is a prolonged risk period for mood and anxiety disorders in this group, with prevalence peaking in early adulthood. This highlights the need for prolonged vigilance and effective targeted interventions in the offspring of depressed parents.

## Graphical abstract

While it is known that parental depression increases risk for offspring depression and anxiety, there are relatively few prospective longitudinal studies following the offspring of depressed parents across the transition from adolescence into adulthood. In a cohort of the offspring of depressed parents followed prospectively for 13 years, the risk period for onset of mood and anxiety disorders was prolonged, highlighting the need for prolonged vigilance in this group who might benefit from early intervention and ongoing support.

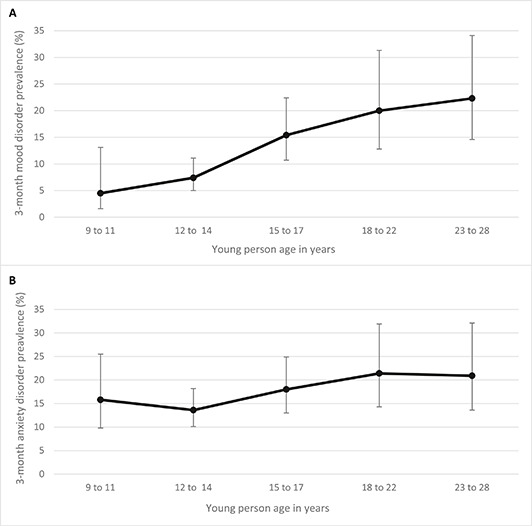

**Key points**

- While it is known that depression in a parent increases risk for offspring depression and anxiety, there are relatively few prospective longitudinal studies following the offspring of depressed parents across the transition from adolescence into adulthood – a key period of risk.
- In a cohort of the offspring of depressed parents followed prospectively for 13 years, the risk period for onset of mood and anxiety disorders was prolonged, extending from childhood to adulthood, with prevalence peaking in adulthood.
- Rates of impairment were high in adulthood, which was influenced by both prior and current mood and anxiety disorders.
- Incorporating family history of depression into routine clinical assessments may help identify individuals who might benefit from early intervention and ongoing support.

## INTRODUCTION

Having a parent with depression is the most common, potent risk factor for anxiety and depression in young people (Lieb et al., 2002; Rice et al., 2017; Weissman et al., 2006). Indeed, approximately half of young people diagnosed with depression have a parent with a history of depression (Brophy et al., 2021). During adolescence, the incidence of many psychiatric disorders including depression and anxiety increases sharply, with the peak period of onset occurring in early adulthood (Kessler et al., 2005). The period between adolescence and young adulthood is, therefore, a critical developmental period for the emergence of psychopathology and involves role and social transitions into adult life (Arnett, 2000; Solmi et al., 2022).

There are very few studies of the offspring of depressed parents that consider the development of mood and anxiety disorders during the transition to adult life. One notable exception are the seminal studies based on the Yale cohort recruited in the early 1980s which have followed‐up high‐risk offspring aged 6–23 years at the initial assessment over a period of 30 years (Weissman et al., 1997, 2006, 2016). These studies have reported lifetime cumulative prevalence rates of disorder measured over very long follow‐up periods and have illustrated high rates of psychopathology in the offspring of depressed parents (Weissman et al., 1997, 2016) as well as poor social functioning (Weissman et al., 2006).

We are not aware of other prospective longitudinal high‐risk studies spanning the transition between adolescence and adulthood. Given that rates of mental health disorder, particularly anxiety and depression, have increased in recent decades (Collishaw, 2015), there is a need to map patterns of development in contemporaneous high‐risk cohorts spanning this transition period. Here we present results from a UK high‐risk cohort recruited in 2007 that makes use of complementary methods to those of the Yale studies and presents data on anxiety, depression and functioning over the transition between adolescence and adult life. We utilise a research design that employs multiple assessments of current psychopathology, as this is useful for describing psychiatric disorder onset, because as well as increasing overall risk for psychopathology in offspring, parental depression may additionally influence the course and severity of psychopathology in offspring over time (Kendler et al., 1999; Lieb et al., 2002; Musliner et al., 2016; Nierenberg et al., 2007; Weissman et al., 2016).

Given the significant investment of effort required to collect multiple waves of detailed data from cohorts over extended periods of time, with some notable exceptions, there is a lack of studies that examine the development of anxiety and depressive disorders from childhood to adulthood in the offspring of depressed parents.

We sought to address the following research aims in a 13‐year prospective study of young people with a parent with recurrent depression:

1. To characterise the prevalence, age at first diagnosis, clinical course and comorbidity profiles for mood and anxiety disorders in the offspring of depressed parents from childhood to adulthood.
2. To examine social functioning, impairment and risky behaviours in the offspring of depressed parents in early adulthood. We additionally tested to what extent adult outcomes were associated with current or past psychopathology.

## METHODS

### Sample

We used data from the Early Prediction of Adolescent Depression (EPAD) study ‐ a prospective, longitudinal UK cohort study of recurrently depressed parents and their offspring. The baseline sample included 337 parents (315 mothers, 22 fathers) with a history of recurrent depression and their offspring (197 females and 140 males aged 9–17 years at baseline assessment). Parents and offspring were assessed via interview and questionnaire over four assessment waves between April 2007 and September 2020. The average length of follow up was 16 months between the first and second waves, 13 months between the second and third, and 8 years between the third and fourth. A flow diagram summarising the study design and rates of participation at each assessment wave is shown in Figure S1. Ethical approval was granted by the Multi‐Centre Research Ethics Committee for Wales and from the School of Medicine Ethics Committee, Cardiff University. Written informed consent and assent were obtained for each participant at each wave. More detail regarding recruitment, assessments and sample characteristics can be found elsewhere (Mars et al., 2012, 2015; Powell et al., 2021).

### Procedure

The sample was recruited primarily from general practices in South Wales. At the time of initial recruitment, parents were screened over the telephone to ensure they met the inclusion criteria: a history of at least two episodes of depression (later confirmed at baseline using diagnostic interview) who had biologically related offspring living at home aged 9–17 years old. Families were excluded if the parent had a diagnosis of bipolar or psychotic disorder at baseline or if the child presented with a moderate to severe learning disability (IQ < 50). If there was more than one eligible child in the household, the youngest child was selected for participation. Therefore, multiple siblings from the same family were not included.

Most assessments took place in the family home with young people and parents interviewed separately. A small number of assessments were undertaken over the telephone or via video‐call as required.

### Measures

#### Young person psychiatric diagnoses

At each of the four assessment waves, a semi‐structured research diagnostic interview, the Child and Adolescent Psychiatric Assessment (CAPA) (Angold & Costello, 2000) or its extension for young adults, the Young Adult Psychiatric Assessment (YAPA) (Angold et al., 1999), was used in separate interviews with parents and offspring to assess offspring DSM‐IV (American Psychiatric Association, 1994) psychopathology in the preceding 3 months. At all phases of the study, all cases (either parent‐reported or young person‐reported) meeting criteria for a psychiatric disorder and cases with subthreshold symptoms were reviewed by two psychiatrists and diagnoses were agreed according to clinical consensus.

The primary outcomes considered in the current paper were any diagnosis of a mood disorder (MDD, dysthymia, bipolar I or II disorder, cyclothymia, adjustment disorder and depressive disorder not otherwise specified) and any diagnosis of an anxiety disorder (generalised anxiety disorder (GAD), social anxiety, separation anxiety, agoraphobia, obsessive compulsive disorder, panic disorder and anxiety disorder not otherwise specified). Throughout the current paper, the reported prevalence and age at first diagnosis of anxiety disorders do not include specific phobias, unless explicitly stated. For both mood and anxiety disorders, additional binary variables were derived for disorder persistence/recurrence, defined as meeting criteria for mood or anxiety disorder more than once across the four (3‐month) assessment waves of the study. Due to the episodic nature of depression, depressive episodes occurring in between assessment waves may have been missed in the current study. Therefore, participants were asked about depressive episodes occurring since the last assessment wave. A life history calendar approach was used to aid recall and thus reduce recall bias (Belli, 1998). As a sensitivity check, proportions of those reporting a depressive episode in between assessment waves were reported.

Comorbidity: Participants were also asked about other disorders and reported on the following sections of the CAPA/YAPA at each phase: attention deficit hyperactivity disorder, oppositional defiant disorder, conduct disorder, disruptive disorder not otherwise specified, bulimia and eating disorder not otherwise specified. Finally, at wave 4, clinical diagnoses of personality disorders (schizotypal and borderline) were made according to clinical consensus. Although personality disorders were not explicitly assessed by standardized interview, in a small number of cases, a personality disorder diagnosis was judged to be the most appropriate diagnosis for the symptoms exhibited. When calculating rates of comorbidity for mood and anxiety disorders, all these disorders were included.

We combined parent and offspring reports of the CAPA diagnoses (either/or) consistent with prior work, recommendations and clinical practice in young people (Angold & Costello, 1995; Mars et al., 2015). At waves 1–3, both parents and offspring completed the CAPA. At the adult follow‐up (wave 4; mean age 23), the young person completed the YAPA, but parents also reported on MDD in the young person using an adapted version of the YAPA. This was done because MDD was a primary focus of the adult follow‐up, so we included parental reports combined with young person reports for consistency with prior assessment phases. Parent and child reports of depression were highly correlated (See Table S1) supporting the validity of this approach in young adults. As parent reports would not typically be used in the assessment of depression in adult mental health services, we conducted a sensitivity analysis calculating mood disorder prevalence at assessment wave 4 using young person report only.

#### Impairment, risky behaviour and functioning in early adulthood

The young people reported on impairment associated with emotional or behavioural problems (Strengths and Difficulties Questionnaire impact supplement (Goodman, 1997)), alcohol use (Alcohol Use Disorders Identification Test (Saunders et al., 1993)), suicide attempt and self‐harm (YAPA), the number of people they could rely on for social support, and education and employment status at wave 4 (see Appendix  S1 for further details on measures).

### Data analysis

To investigate patterns of mood disorders and anxiety over the course of the study, we first generated the 3‐month prevalence of mood and anxiety disorders at each of the four assessment waves. As the offspring were different ages when they started the study, to investigate patterns by developmental phase, we then calculated the 3‐month prevalence of mood and anxiety disorders by participant age in years. To ensure sufficient numbers of participants for each age, we pooled age in years into the following age groups: childhood (9–11 years; *n* = 177), early adolescence (12–14 years; *n* = 414), late adolescence (15–17 years; *n* = 275), the transition from adolescence to adulthood (18–22 years; *n* = 94) and the mid‐to‐late twenties (23–28 years; *n* = 90). To estimate disorder prevalence by age group, we used the ‘glm’ command on Stata v17, and clustered on participant identification number to account for participants who may have been included in the same age group more than once (e.g., were within the same age group for two assessment waves). Table S2 shows which of the four assessment waves contribute to each age group. Estimates of prevalence in the twenties (wave 4) may be less robust due to lower numbers resulting from the longer lag and associated participant dropout between waves 3 and 4. We therefore investigated differences between participants who participated in waves 1–3 only compared to those who participated in wave 4 (see Table S3) and accounted for potential bias due to missing data by applying inverse probability weighting (IPW; see Appendix S2) to estimations of disorder prevalence by pooled age groups (Table S4; Seaman & White, 2013). Social outcomes in early adulthood focussed on the fourth assessment phase only (mean age 23, range 18–28). We also tested whether these outcomes were associated with current or previous (waves 1–3) mood or anxiety disorders using logistic regression with IPW applied.

## RESULTS

### The 3‐month prevalence of mood and anxiety disorders from childhood to early adulthood

**FIGURE 1:**
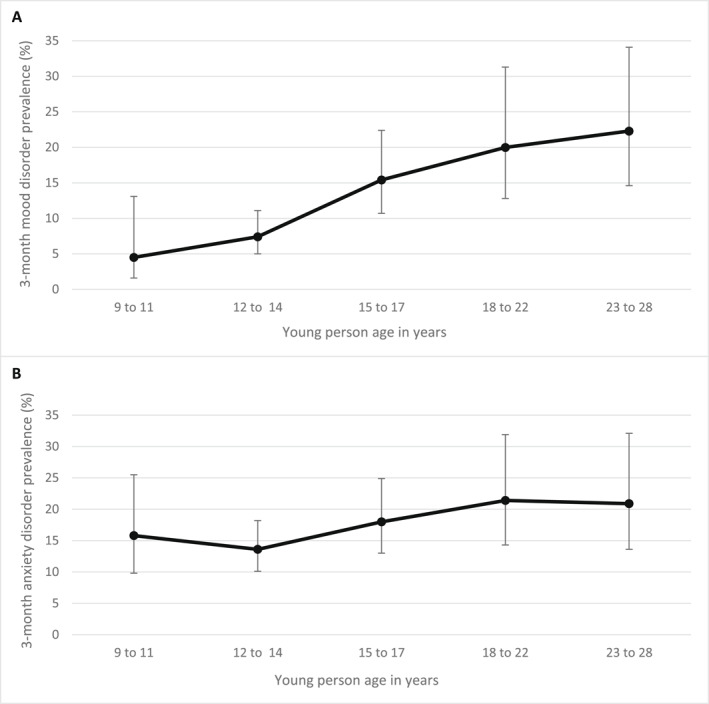
Rates of mood and anxiety disorders by age group. The 3‐month prevalence of any mood disorder (A) and anxiety disorder (B) are shown. The error bars show the 95% confidence intervals. Inverse probability weighting (IPW) was applied. *n* = 177 at 9–11 years; *n* = 414 at 12–14 years; *n* = 275 at 15–17 years; *n* = 94 at 18–22 years; *n* = 90 at 23–28 years. IPW, inverse probability weighting.

Of the total sample (*n* = 337), 24.9% met DSM‐IV criteria for a current (3‐month) mood disorder and 33.2% for a current (3‐month) anxiety disorder at least once during the four assessment waves. Prevalence was twice as high in females as in males. When including specific phobias, the prevalence of anxiety disorder increased to 39.3%. We next investigated the 3‐month prevalence of mood and anxiety disorders by age group as described in the methods section (childhood 9–11 years; early adolescence 12–14 years; late adolescence 15–17 years; the transition between adolescence and adulthood 18–22 years; and the mid‐to‐late twenties 23–28 years). Figure 1 shows the 3‐month prevalence by age group of mood disorders (A) and anxiety disorders (B).

The 3‐month prevalence of mood disorder increased with age from 4.5% (95% CI 1.6–13.1) during childhood (ages 9–11) to 22.3% (95% CI 14.6–34.1) by the mid‐to‐late twenties (ages 23–28). Figure 1A shows a peak in the 3‐month prevalence of mood disorder at the transition to adulthood and during the mid‐to‐late twenties.

In terms of the sex of those affected by mood disorders, although confidence intervals overlapped between males and females for all age groups, we did observe some patterns suggesting sex differences in prevalence by age group (Table 1), consistent with epidemiological research. As expected, in childhood, the proportion of affected males was higher than for females, while in early and late adolescence, there was an excess of affected females compared to males. At the transition to adult life and in the mid‐to‐late twenties however, there were more affected males than females. While the 3‐month prevalence of mood disorder generally increased in females from early adolescence onwards, the 3‐month prevalence in males remained relatively low until the transition to adult life, when rates increased and remained high into the mid‐to‐late twenties. Thus, the rise in 3‐month prevalence of mood disorders at the transition to adulthood was particularly pronounced in males.

**TABLE 1:** Prevalence of mood and anxiety disorders by age group in males compared to females.

Disorder	Age group	Prevalence	
		Males % (95% CI)	Females % (95% CI)
Any mood	Childhood (9–11 years)	6.9 (1.5–32.7)	2.9 (0.9–8.7)
	Early adolescence (12–14 years)	4.8 (2.2–10.6)	9.6 (6.1–15.2)
	Late adolescence (15–17 years)	6.7 (3.2–14.1)	20.5 (13.6–30.8)
	Transition to adulthood (18–22 years)	24.0 (11.4–50.5)	17.9 (10.3–30.9)
	Mid to late twenties (23–28 years)	23.1 (12.1–44.1)	21.7 (12.3–38.2)
Any anxiety	Childhood (9–11 years)	20.3 (9.6–42.9)	12.7 (7.3–22.2)
	Early adolescence (12–14 years)	8.7 (5.0–15.0)	17.7 (12.6–25.1)
	Late adolescence (15–17 years)	17.7 (10.2–30.7)	18.2 (12.2–27.2)
	Transition to adulthood (18–22 years)	18.8 (8.8–39.8)	22.8 (14.2–36.6)
	Mid to late twenties (23–28 years)	22.5 (11.7–43.3)	19.7 (11.1–34.7)

*Note*: The 3‐month prevalence of any mood disorder and anxiety disorder are shown. IPW was applied. *n* = 177 at 9–11 years; *n* = 414 at 12–14 years; *n* = 275 at 15–17 years; *n* = 94 at 18–22 years; *n* = 90 at 23–28 years.

Abbreviations: CI, confidence interval; IPW, inverse probability weighting.

For anxiety disorder, the 3‐month prevalence during childhood was 15.8% (95% CI 9.8%–25.5%). Thus, as expected, the 3‐month prevalence of anxiety disorders was higher in childhood compared to the 3‐month prevalence of mood disorders. The 3‐month prevalence of anxiety disorders continued to be high through adolescence (13.6% (95% CI 10.1–18.2) at ages 12–14; 18.0% (95% CI 13.0–24.9) at ages 15–17) and peaked during the transition from adolescence to adulthood at ages 18–22 (21.4%, 95% CI 14.3–31.9), as shown in Figure 1B.

Similar to mood disorders, confidence intervals overlapped between males and females for all age groups, though we did observe patterns suggestive of gender differences (Table 1). While anxiety disorders in childhood appeared to be more common in males than females, throughout adolescence and the transition to adult life, they were more common in females than in males. During the mid‐to‐late twenties, anxiety disorders were more common in males than females.

Results for the most common mood disorder (MDD) and anxiety disorder (GAD) in the study are shown in Figure S2. Patterns of MDD prevalence were very similar to those observed for mood disorders overall. Patterns of GAD prevalence were similar to those observed for anxiety disorders, though prevalence of GAD was low in childhood (3.6%, 95% CI 1.5–8.9) and increased with age.

Results were broadly similar when analysing the data by assessment phase rather than by age, that is, rates increased with successive assessment phases, consistent with increases with successive age groups (see Table S5).

### Age at first diagnosis, clinical course and patterns of comorbidity of mood and anxiety disorders in the offspring of depressed parents

The mean age at first diagnosis for mood disorders was 16.5 years (range 9–26). The mean age at first diagnosis for mood disorders was later in males (17.5 years) than females (16.1 years). The mean age at first diagnosis for anxiety disorders (excluding phobias) was 14.5 years (range 9–28). The mean age at first diagnosis for anxiety disorders was similar for males (14.0 years) and females (14.7 years). When including phobias, the mean age at first diagnosis for anxiety disorders was 14.4 years (range 9–28). Wide ranges in age at first diagnosis were observed for both mood and anxiety disorders, including some very early‐onset cases. For example, for MDD, the lowest age at first diagnosis was 10 years. Age at first diagnosis for all mood and anxiety disorders examined are shown in Figure S3.

Across the four study assessments, 7.2% of the sample had more than one episode of mood disorder. 13.3% of the sample met criteria for anxiety disorder at more than one assessment wave.

**FIGURE 2:**
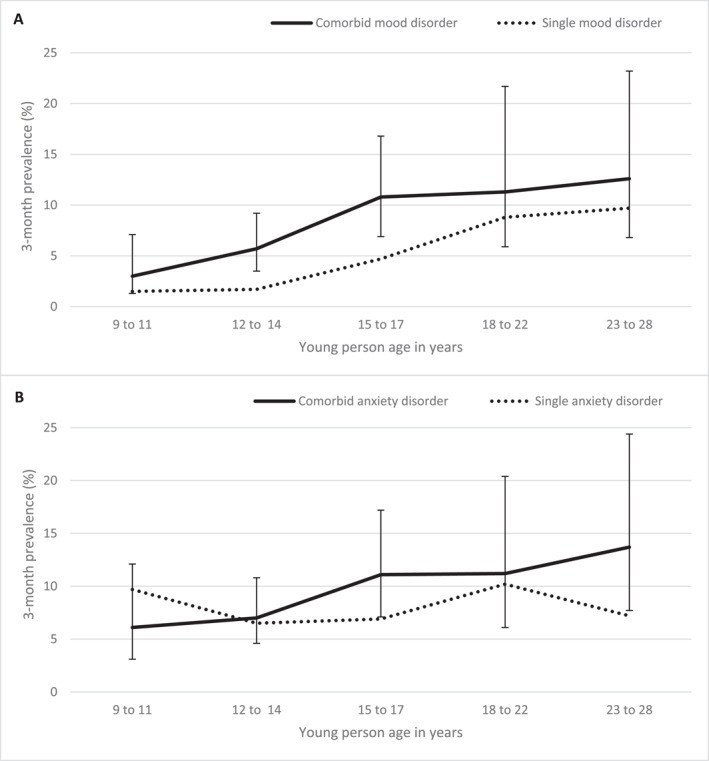
Rates of comorbidity within mood and anxiety disorders by age group. The 3‐month prevalence of single mood disorder versus comorbid mood disorder (A), and single anxiety disorder versus comorbid anxiety disorder (B) are shown. The error bars show 95% confidence intervals for the comorbid disorder prevalence estimates. Inverse probability weighting (IPW) was applied. *n* = 177 at 9–11 years; *n* = 414 at 12–14 years; *n* = 275 at 15–17 years; *n* = 94 at 18–22 years; *n* = 90 at 23–28 years. IPW, inverse probability weighting.

We assessed rates of comorbidity, defined as having two or more disorders concurrently. 18.1% of individuals had a mood disorder that was concurrently diagnosed with another psychiatric disorder at least once over the study assessments. The figure for anxiety disorders was 20.7%. In those individuals with a mood disorder, nearly three quarters (72.7%) had a comorbid psychiatric disorder concurrently. For anxiety, the figure was 62.3%. Concurrent mood and anxiety disorder was the most common form of disorder comorbidity. Details on the cumulative prevalence and types of comorbidity are shown in Figure S4. Figure 2 shows the prevalence of comorbidity in mood and anxiety disorders plotted by age‐group. In general, comorbid mood disorder was more common than a single mood disorder (Figure 2A). For anxiety, while single disorders were common in childhood, comorbidity increased thereafter (Figure 2B).

### Social functioning, impairment, and risky health behaviours in early adulthood

In early adulthood (wave 4; age range 18–28), half of the overall sample reported having emotional or behavioural problems that caused distress or interfered with their ability to function in aspects of everyday life (Table 2). Ten percent reported a recent suicide attempt or deliberate self‐harm.

**TABLE 2:** Rates of social, health, education and occupational outcomes in young adults at most recent assessment wave (mean age 23 years; range 18–28).

Outcome	% (95% CI)	F:M
Not in education, employment or training (NEET)	14.9 (11.0–19.4)	1.4:1
Completed degree/currently in university	59.4 (53.6–65.0)	1.9:1
Poor social support (only one person or no one to rely on)	23.6 (18.9–28.8)	1:1.2
Harmful alcohol use	31.4 (26.2–37.0)	1:1.1
Recent suicide attempt or deliberate self‐harm	9.6 (6.5–13.5)	1.2:1
SDQ impact of emotional or behavioural problems on daily functioning = borderline or abnormal	52.3 (46.9–57.7)	1.6:1

*Note*: The rates of social and health outcomes in the young people at the most recent assessment wave are reported (wave 4; *n* = 144). IPW was applied.

Abbreviations: CI, confidence interval; F:M, female:male ratio; IPW, inverse probability weighting; SDQ, strengths and difficulties questionnaire.

For educational, occupational and social adult outcomes, 59.4% of young people had completed a degree or were currently studying at university, while 14.9% were currently not in education, employment or training (NEET) (Table 2). A quarter of the young people reported having no one or only one person to rely on for social support. Over 30 percent reported harmful alcohol use.

Both previous and current mood and anxiety disorders were associated with impairment in daily functioning and with not completing a degree or attending university in early adulthood (Table 3). Only current mood and anxiety disorders were associated with NEET status and recent self‐harm or suicide attempt. Neither previous nor current mood or anxiety disorders were associated with poor social support or harmful alcohol use.

**TABLE 3:** Influence of prior versus current mood and anxiety disorders on social outcomes in early adulthood at most recent assessment wave (mean age 23 years; range 18–28).

Outcome	Previous mood or anxiety disorder association with outcome (OR, 95% CI, *p*‐value)	Current mood or anxiety disorder association with outcome (OR, 95% CI, *p*‐value)
Not in education, employment or training (NEET)	0.77, 0.38–1.58, 0.476	**5.68, 2.79–11.54, <0.001**
Completed degree/currently in University	**0.41, 0.25–0.68, <0.001**	**0.50, 0.31–0.81, 0.005**
Poor social support	1.10, 0.61–1.97, 0.751	1.07, 0.61–1.88, 0.808
Harmful alcohol use	0.66, 0.38–1.15, 0.142	0.87, 0.51–1.46, 0.584
Recent suicide attempt or deliberate self‐harm	2.01, 0.93–4.41, 0.078	**5.31, 2.25–12.57, <0.001**
SDQ impact of emotional or behavioural problems on daily functioning – borderline or abnormal	**2.98, 1.79–4.96, <0.001**	**8.67, 4.67–16.10, <0.001**

*Note*: Unadjusted associations between previous mood or anxiety disorder (any anxiety or mood disorder at assessment waves 1–3) and social outcomes in early adulthood (assessment wave 4), in addition to associations between current (wave 4) mood or anxiety disorder and social outcomes in early adulthood, are shown. Statistically significant results are highlighted in bold. IPW was applied. Results remained similar when current and prior disorders were entered simultaneously.

Abbreviations: CI, confidence interval; IPW, inverse probability weighting; OR, odds ratio; SDQ, strengths and difficulties questionnaire.

### Sensitivity analyses

We undertook two sensitivity checks. First, we restricted analyses to young person reports only at wave 4 (mean age 23; range 18–28) for mood disorder and found that results remained similar (Figure S5). Second, we found that the cumulative prevalence of MDD increased by 9.8% from 17.8% to 27.6% when depressive episodes occurring in between waves were considered (see Table S6), suggesting that our estimates of mood disorder prevalence may be conservative. We also used IPW to adjust results for potential bias arising from missing data. Unweighted results (without IPW to adjust for missing data bias) are shown in Table S4.

## DISCUSSION

To our knowledge, this is the first UK prospective longitudinal study of the offspring of depressed parents to track the prevalence and investigate the characteristics of mood and anxiety disorder from childhood to adulthood with multiple assessment timepoints, and to investigate links with functioning in emerging adulthood. The cumulative prevalence of mood (24.9%) and anxiety disorder (33.2%; 39.3% when specific phobia was included) across the four assessment points of the present study are substantially higher than those reported in this age group in the general population. For example, a population cohort study using an accelerated longitudinal design and the same diagnostic measure used in the current study (CAPA) to estimate the 3‐month prevalence of mood and anxiety disorders, found that the cumulative prevalence of mood and anxiety disorders across ages 9–21 were 12% for mood disorder and 17% for anxiety disorder (Copeland et al., 2011). It is noteworthy that the 3‐month prevalence of mood disorder during childhood and adolescence observed in the current study are also greatly increased compared to those from the general population (Costello et al., 2003).

When investigating disorder prevalence by age‐group, results highlighted a prolonged risk period for mood and anxiety disorders extending into the mid‐twenties, with some very early‐onset cases in childhood and rates of affected individuals increasing through adolescence and into adulthood. The transition to adult life was a key period when prevalence of these disorders rose substantially. Interestingly, the peak in prevalence for anxiety disorders was only slightly earlier in adulthood than for mood disorders. The epidemiology of anxiety disorders commonly involves onset in childhood but there is strong developmental patterning depending on the types of anxiety disorder examined (Merikangas et al., 2010). For instance, Weissman et al. (2006) observed a peak in anxiety incidence before the age of 10, far earlier than the peak in anxiety prevalence observed in the current study. Weissman et al. (2006) included simple phobias in their anxiety disorder estimates, which typically onset earlier in childhood. However, including specific phobias made minimal difference to the estimates of age at first diagnosis of anxiety disorder in the current study. It should be noted that due to the different methods of assessment (lifetime disorder since last assessment vs. current disorder), the estimated rates of disorder reported in Weissman et al. (2006) are not directly comparable to those reported here.

The rise in prevalence of mood and anxiety disorders at the transition to adulthood observed in the current study was particularly marked in males. In females, prevalence began to increase from early adolescence and was higher than in males throughout adolescence. In males, rates remained lower in adolescence before rising substantially to higher than the prevalence in females at the transition to adult life (ages 18–22). This is somewhat consistent with previous work on the offspring of depressed parents where Weissman and colleagues found that the peak period of risk for MDD was 15–20 regardless of familial risk, with evidence that this risk period extended up to the age of 30 for males (Weissman et al., 2006). There is also evidence from community samples suggesting that the female excess for depression reduces during the mid‐twenties (Patton et al., 2014). Coupled with previous evidence, the current findings may suggest a later onset for mood and anxiety disorders in males compared to females. Previous work has observed a substantial drop in levels of service use during young adulthood compared to adolescence, with males less likely to be receiving help in early adulthood than females (Copeland et al., 2015). Monitoring young adults, particularly men, with a family history of depression for emerging mood and anxiety problems may be important to consider clinically.

Age at first diagnosis varied widely in this study, with onset at a very young age observed in a small number of cases (mood disorder onset range = 9–26 years). This finding bears resemblance to a previous study of the offspring of depressed parents that found that though mean age of onset was similar to a control group, the range in age of onset was much wider, and very early‐onset cases were observed only in those with a family history of depression (Weissman et al., 2016). In the current study, we found that the 3‐month prevalence of MDD during childhood was 3%, which is high in comparison to longitudinal population studies using similarly stringent measures of diagnosis prevalence (Costello et al., 2003).

In terms of recurrence/persistence, approximately 7% of young people in the study experienced multiple mood disorder episodes and 13% met criteria for an anxiety disorder more than once. An onset for depressive disorders during adolescence has been associated with a more persistent and relapsing course of symptomatology in community samples (Weavers et al., 2021). A previous, small study found tentative evidence of an increased 2‐year depression recurrence rate in the offspring of depressed parents compared to controls (Warner et al., 1992) and increased rates of MDD recurrence in high‐risk offspring were also reported in Weissman et al. (2016). Data from different study designs also highlights that parental depression is associated with a prolonged treatment trajectory, implying persistence, in individuals with mood disorder (Musliner et al., 2016). Preventing persistence or recurrence of mood and anxiety disorders in the offspring of depressed parents may be important to consider. Indeed, the depression prevention programme with the smallest number needed to benefit observed to date (Hetrick et al., 2016) specifically focussed on the offspring of depressed parents and also included a large proportion of youth with a prior history of depressive disorder (Garber et al., 2009).

In the current study, we observed high comorbidity, with concurrent mood and anxiety disorder representing the vast majority of cases of disorder comorbidity, consistent with findings from the general population (Costello et al., 2003). Studies of *adults* have shown that a family history of depression is associated with an earlier age at onset of depression, greater recurrence and greater comorbidity (Kendler et al., 1999; Lieb et al., 2002; Musliner et al., 2016; Nierenberg et al., 2007; Weissman et al., 2016). The current study extends those findings in adults by highlighting that these characteristics of familial depression are evident much earlier in life.

We assessed impairment and functioning in early adulthood and found that over half of the sample reported impairment in daily functioning. Almost a quarter of the present sample lacked a social support network. A previous study found social functioning scores to be worse in offspring of depressed parents compared to controls (Weissman et al., 1997). Prevalence of self‐harm/suicide attempt (9.6%) and harmful alcohol use (31.4%) were higher in the current study than reported in early adulthood in the general population, though estimates vary between studies (Copeland et al., 2012; Fernandes et al., 2020; Foley et al., 2006; Kessler et al., 2005). Our estimates are also high when compared to a previous study of the offspring of depressed parents that reported lifetime, rather than current, cumulative rate estimates of 16% for suicide attempts or gestures and 15% for alcohol abuse (Weissman et al., 1997). Over half (59.4%) of the current sample had a degree or were in university and 14.9% were NEET, similar to the 11% of the UK population estimated to be NEET by the Office for National Statistics (Watson, 2019). Though a previous study in the Yale cohort similarly found no significant difference between offspring of depressed parents and controls in terms of employment status and level of education (Weissman et al., 1997), the finding contrasts to evidence from national datasets of parental depression having a negative impact on offspring educational outcomes (Brophy et al., 2021; Shen et al., 2016). Though estimates in the current study are inverse probability weighted to account for potential bias arising from missing data, it should be noted that parent education at baseline was associated with drop out by the final assessment wave (See Table S3). When investigating the association of prior or current mood or anxiety disorders in the offspring with social outcomes in adulthood, we found that both prior and current mood or anxiety disorder were associated with impairment in daily functioning and not attending university or attaining a degree. Only current mood or anxiety disorder was associated with NEET status and with self‐harm or suicide attempt in adulthood. Given the elevated risk of mood and anxiety disorder in the offspring of depressed parents (Lieb et al., 2002; Rice et al., 2017; Weissman et al., 2006), these findings highlight the need to consider both concurrent and long‐term impacts of mood and anxiety disorder on functioning and social outcomes in adulthood in this high‐risk group. Neither prior nor current mood or anxiety disorder were associated with alcohol use or social support in adulthood. However, social support is a predictor of future depression (Kendler et al., 2005), and therefore could play a role in mood disorder recurrence in this population.

Strengths of this study include use of a large cohort of the children of depressed parents followed prospectively over 13 years that includes key developmental phases using detailed clinical measures from multiple informants. Limitations of the present study include that prevalence estimates may be conservative as we focussed on assessing current (3‐month) psychopathology. We chose to focus on current psychopathology given evidence that this can yield more reliable assessments than relying on longer windows of retrospective reporting where participants are more likely to forget past symptoms and experiences (Hardt & Rutter, 2004; Wells & Horwood, 2004). As it is possible that depressive episodes occurring in between assessment waves may have been missed in the current study, participants were asked about depressive episodes occurring since the last assessment wave. When episodes reported between waves were considered, the prevalence of depressive episodes increased, suggesting that our estimates may be conservative. It stands to reason that the age of first diagnosis cannot be less than the age of the youngest participants at the study baseline (i.e. 9 years). This seems unlikely to impact substantially on age at first onset estimates given that the median estimates of first onsets from community studies are 11 for anxiety and 30 for depressive disorder (Kessler et al., 2005). We analysed cohort data from four assessment phases as an accelerated longitudinal design and accounted for clustering of participants. This makes an assumption that participants who are in the same age group at different assessment waves are equivalent and ‘exchangeable’, and that there is a lack of systematic differences between, for example, participants who were aged 9–11 years at assessment wave 1 to participants who were aged 9–11 years at assessment wave 2. This assumption would imply the absence of interaction between assessment wave effects and developmental change (i.e. age‐group). We found no evidence of an interaction between assessment wave and age in predicting mood or anxiety disorder, thereby supporting our use of this approach (Table S7). Nonetheless, it is possible that external events occurring during the course of the data collection period may impact on exchangeability of individuals of similar ages at different assessment waves, for example, UK government austerity cuts that were implemented between the first three assessment waves (2007–2012) and the fourth assessment wave (2017–2020). As fourth wave data collection was completed for approximately 95% of participants before the covid pandemic, this is unlikely to affect results. Future studies may be able to harness complex statistical methods to more thoroughly test for and adjust for any potential cohort effects. Another limitation is the changing denominator when looking at disorder prevalence by age group in years. As this study was conducted across four assessment waves with children aged 9–17 at baseline, when reporting disorder prevalence by age, the sample size of some age groups is smaller than others. Disorder prevalence in the twenties (wave 4), for example, may be less robust due to the longer lag and resulting participant dropout between waves 3 and 4. However, broad patterns of disorder prevalence by age group are similar to what is observed in the wave‐by‐wave format (See Table S4). We applied IPW to all reported results to account for potential bias arising from missing data (Seaman & White, 2013). Estimates following weighting were slightly increased compared to results before weighting (See Table S4), suggesting that unweighted results may have been slight underestimates of the true rate of disorders. Prevalence of psychotic symptoms was not reported in the current study, though this may be a marker of severity of mood and anxiety disorder (Stochl et al., 2015). However, a prevalence of 8.4% for reported psychotic experiences in the offspring of depressed parents from the same study sample has been reported previously (Bevan Jones et al., 2016).

## CONCLUSION

To conclude, in a prospective study of the offspring of depressed parents spanning 13 years, as expected, we found that prevalence of mood and anxiety disorders were substantially higher than those reported in comparable age groups in the general population, but also showed an extended period of risk from childhood to early adulthood. Mood and anxiety disorders were often comorbid. Using complementary methods to those used by a US cohort of offspring of depressed parents recruited in the 1980s, the current study tracked the transition period from childhood and adolescence to adulthood prospectively over four assessment timepoints in a UK cohort of the offspring of depressed parents. The transition from adolescence to adulthood was a key emerging point for mood and anxiety disorders, particularly in males. These young people demonstrated high rates of poor social outcomes in early adulthood, with over half reporting impairment in daily functioning. Impairment and educational outcomes in adult life were associated with both prior and current mood or anxiety disorder. These findings suggest that incorporating family history of depression into routine clinical assessments may help identify who should be considered for early intervention and continued support – a recommendation of the World Health Organisation (World Health Organization (WHO), 2013) which may circumvent poor adult outcomes (Merry et al., 2011).

## AUTHOR CONTRIBUTIONS

**Victoria Powell:** Conceptualization, Data curation, Formal analysis, Investigation, Visualization, Writing – original draft. **Jessica Lennon:** Conceptualization, Data curation, Formal analysis, Investigation, Visualization, Writing – original draft. **Rhys Bevan Jones:** Writing – review & editing. **Alice Stephens:** Writing – review & editing. **Bryony Weavers**: Writing – review & editing. **David Osborn:** Funding acquisition, Writing – review & editing. **Judith Allardyce:** Writing – review & editing. **Robert Potter**: Writing – review & editing. **Ajay Thapar:** Funding acquisition, Writing – review & editing. **Stephan Collishaw:** Funding acquisition, Writing – review & editing. **Anita Thapar:** Funding acquisition, Writing – review & editing. **Jon Heron:** Funding acquisition, Writing – review & editing. **Frances Rice:** Conceptualization, Funding acquisition, Project administration, Supervision, Writing – review & editing.

## CONFLICT OF INTEREST STATEMENT

The authors have declared that they have no competing or potential conflicts of interest.

## ETHICAL CONSIDERATIONS

Ethical approval was granted by the Multi‐Centre Research Ethics Committee for Wales and from the School of Medicine Ethics Committee, Cardiff University. Written informed consent and assent were obtained for each participant at each wave.

## Supporting information

Supplementary Material

## Data Availability

Due to ethical restrictions, data collected at assessment waves 1 to 3 cannot be made openly available. Supporting data collected at assessment wave 4 is openly available from the Cardiff University data repository at http://doi.org/10.17035/d.2023.0263728184.
