# Supplementary material for: Following the children of depressed parents from childhood to adult life: A focus on mood and anxiety disorders
Source: JCPP Adv. 2023 Jun 18;3(4):e12182. doi: 10.1002/jcv2.12182 (PMC10694536; doi:10.1002/jcv2.12182)
Supplement: Supplementary file 1 — Supplementary Material [file JCV2-3-e12182-s001.docx]

**Supporting Information**

**Wave 1**: 337 families (recurrently depressed adults and their children) recruited that meet inclusion criteria at baseline

*Average length of follow up = 16 months*

**Wave 2**: 319 families retained (94.7% of baseline sample)

*Average length of follow up = 13 months*

**Wave 3**: 309 families retained (91.7% of baseline sample)

***73 families of the baseline sample (337) were not contactable at wave 4:***

*41 withdrew before wave 4*

*19 withdrew during wave 4*

*4 were lost to follow up / no working contact details*

*9 did not wish to participate this time round (due to illness, bereavement or busy work schedules) but consented to future contact*

*Average length of follow up = 8 years*

**Transition period from adolescence to adulthood**

**Wave 4**: 197 families retained (58.5% of baseline sample; *74.6% of the 264 families who were contactable for participation at wave 4*)

**Figure S1. Retention of the baseline sample across waves 1 to 4 of the EPAD study.** The Early Prediction of Adolescent Depression (EPAD) study was conducted between April 2007 and September 2020 in four assessment waves. Families who participated at each wave via questionnaire, interview or both are reported. Only 264 families were contactable at wave 4 due to loss of up-to-date contact details, withdrawal from the study, death and declining to participate due to ill health, bereavement or other commitments such as work (n=73). Of the 264 contactable families at wave 4, 67 were unresponsive despite multiple communication attempts. Of these 67, 48 families were contacted 3 or more times with no response, 2 of the parents had died and there was no response from the offspring, and 17 families initially responded to contact but ultimately did not commit to taking part. Of the 17 who provided details, the most common reason for being unable to commit to taking part was being too busy, namely with work or life events (e.g. moving house)

**Table S1. Correlation between parent and young person reports of depression symptoms and SDQ impact score**

| **Parent and child report correlations** | **r (95% CI)** | **p** |
| --- | --- | --- |
| Depression symptoms wave 1 | .49 (0.40 - 0.57) | <0.001 |
| Depression symptoms wave 2 | .36 (0.25 - 0.46) | <0.001 |
| Depression symptoms wave 3 | .47 (0.37 - 0.56) | <0.001 |
| Depression symptoms wave 4 | .50 (0.36 - 0.61) | <0.001 |
| SDQ impact of emotional or behavioural problems on daily functioning wave 4 | .39 (0.24 - 0.52) | <0.001 |

Pearson correlations between young person (self) and parent reports were calculated for measures where self and parent reports were combined using an either/or approach. *CI* confidence interval, *SDQ* strengths and difficulties questionnaire

**Appendix S1. Measures for impairment, risky behaviour and functioning outcomes in early adult life**

*Education and employment:* Young people reported on their education and employment via questionnaire. A binary variable (0=no, 1=yes) capturing whether the young person was not currently in education, employment or training (NEET status) was derived. An additional binary variable for whether the young person had completed a degree or was currently in university was derived.

*Distress and impairment:* Young person and parent-reports on the impact supplement of the Strengths and Difficulties Questionnaire (SDQ) were used to assess distress and impairment (at home, school, in friendships or in leisure activities) associated with mental health problems (Goodman, 1997). Five items with responses of “Not at all” (0), “Only a little” (0), “A medium amount” (1) or “A great deal” (2) were summed to give a maximum total score of 10. Those scoring 1 were classed “borderline” and those scoring 2 or more were classed as “abnormal” as recommended previously. Child and parent-reported “borderline” or “abnormal” scores were combined using an either/or approach. Parent and child reports were highly correlated (Supplement 2).

*Suicide or self-harm:* As part of the YAPA (Angold et al., 1999) during wave 4 interviews, the young people reported whether they had recently self-harmed or attempted suicide, from which a binary variable was derived.

*Social support:* As part of the interview at wave 4, the young people were asked to list the people they could most rely on for social support. From this, a binary variable was derived for those with only one or no people to rely on, versus those with two or more people they could rely on.

*Harmful alcohol use:* The self-reported Alcohol Use Disorders Identification Test (AUDIT) – a reliable and valid measure of harmful alcohol use (Saunders et al., 1993) – was used during interview at wave 4. The AUDIT consists of 10 items each rated on a 5-point scale from 0 to 4 and is summed to give a maximum total score of 40. Those meeting the validated threshold for the AUDIT by scoring 8 or more were classed as having harmful alcohol consumption levels, as recommended previously.

**Table S2. The contribution of each assessment wave to each age group**

|  | **Age group** | | | | |
| --- | --- | --- | --- | --- | --- |
| **Assessment wave** | **9-11 years n** | **12-14 years n** | **15-17 years n** | **18-22 years n** | **23-28 years n** |
| **1** | **123** | **149** | **62** | **0** | **0** |
| **2** | **45** | **142** | **96** | **6** | **0** |
| **3** | **9** | **123** | **117** | **34** | **0** |
| **4** | **0** | **0** | **0** | **54** | **90** |
| **Total** | **177** | **414** | **275** | **94** | **90** |

For each age group in years, the number of participants from assessment waves 1, 2, 3 and 4 is shown.

**Table S3. Comparison of young people who participated at wave 4 versus those who did not**

|  | **Young people who participated at wave 4 (%)** | **Young people who did not participate at wave 4 (%)** | **T-test/Chi-squared p-value** |
| --- | --- | --- | --- |
| ***Baseline*** | | | |
| **Age** | Mean 12.4 (SE 0.2) | Mean 12.3 (SE 0.2) | .705 |
| **Female** | 107 (60.8) | 90 (55.9) | .362 |
| **Any mood disorder** | 6 (3.4) | 10 (6.2) | .227 |
| **Any anxiety disorder** | 20 (11.4) | 19 (11.8) | .900 |
| **Any behavioural disorder** | 9 (5.1) | 15 (9.3) | .134 |
| **ADHD** | 1 (0.6) | 2 (1.2) | .511 |
| **Any disorder*** | 33 (18.8) | 39 (24.2) | .221 |
| **Single parent household** | 42 (23.9) | 55 (34.2) | **.037** |
| **Family income <£20,000*** | 41 (24.4) | 59 (44.0) | **<.001** |
| **Parent has no qualifications of GCSE level or above*** | 14 (8.0) | 31 (19.3) | **.002** |
| **Parent severe depression ever** | 57 (32.4) | 39 (24.2) | .104 |
| ***Wave 3*** | | | |
| **Any mood disorder** | 19 (11.0) | 11 (9.7) | .724 |
| **Any anxiety disorder** | 23 (13.4) | 15 (13.3) | .981 |
| **Any behavioural disorder** | 10 (5.8) | 14 (12.4) | .051 |
| **ADHD** | 4 (2.3) | 1 (0.9) | .365 |
| **Any disorder** | 52 (30.2) | 30 (26.5) | .502 |
| **Single parent household** | 60 (35.9) | 49 (45.8) | .104 |
| **Family income <£20,000** | 45 (26.5) | 60 (49.2) | **<.001** |
| **Parent has no qualifications of GCSE level or above** | 25 (14.8) | 23 (18.9) | .357 |
| **Parent severe depression ever** | 58 (33.0) | 39 (24.2) | .077 |

To investigate patterns of missing data and predictors of missingness at wave 4, a number of baseline measures of psychiatric, social and demographic factors were compared between those who did participate at wave 4 and those who did not. The same analysis was conducted using equivalent measures from the previous assessment wave (wave 3) rather than baseline measures. This was conducted to assess whether young people participating at wave 4 differed compared to those who did not participate according to baseline measures, or measures at a more recent wave (wave 3), or both. In other words, we tested whether attrition since the last assessment wave differed to attrition at any point in the study. *ADHD* attention deficit/hyperactivity disorder, *GCSE* general certificate of secondary education. * indicates that the variable was used in a logistic model to derive inverse probability weights as detailed in Supplement 6

**Appendix S2. Inverse probability weighting (IPW)**

Inverse probability weighting (IPW) to was used to investigate potential bias due to non-random missing data. IPW involves weighting complete cases by the inverse probability of their being missing. To generate weights, a logistic model was specified in the full sample (n=337) using baseline measures of whether or not the parent had any qualifications of GCSE level or above, whether or not the family income was less than £20,000, and presence of disorder in the child at baseline. Parent education and family income at baseline were included as they both predicted missingness (non-participation in the interviews and questionnaires) in the analysis sample at wave 4 (Supplement 5). Presence of disorder in the child at baseline was included as this was related to the study outcomes. Minimal missing data on indicators used to derive weights were singly imputed as the modal value (all indicators had <12% missing data). The Hosmer-Lemeshow test was used to assess the fit of the missingness model, with results showing no indication of poor fit (Hosmer-Lemeshow χ2(3)=4.042, p=0.400). Weights ranged from 1.71 to 4.47. The distribution of weights was positively skewed, but truncation of the right-hand tail made no difference to weighted results. Results using non-truncated weights are presented in this paper. The distribution of weights was similar in those who participated at wave 4 and those who were missing at wave 4. There were no zero-fitted probabilities in complete or incomplete cases. Rates of mood disorders and anxiety disorders were re-calculated with the weights were applied. Rates increased slightly when the weights were applied, suggesting that disorder prevalence may have been underestimated at wave 4 (Supplement 7).

**Table S4. Comparison of rates of disorders in young people with and without inverse probability weights applied**

|  | **Disorder prevalence % (95 CI)** | | | |
| --- | --- | --- | --- | --- |
| **Age group in years** | ***Any mood disorder*** | | ***Any anxiety disorder (excluding phobia)*** | |
|  | ***Before IPW*** | ***IPW applied*** | ***Before IPW*** | ***IPW applied*** |
| 9-11 | 4.0 (1.5 – 10.6) | 4.5 (1.6 – 13.1) | 11.4 (7.3 – 17.8) | 15.8 (9.8 – 25.5) |
| 12-14 | 6.5 (4.4 – 9.7) | 7.4 (5.0 – 11.1) | 12.3 (9.2 – 16.4) | 13.6 (10.1 – 18.2) |
| 15-17 | 13.1 (9.1 – 18.8) | 15.4 (10.7 – 22.4) | 15.6 (11.5 – 21.3) | 18.0 (13.0 – 24.9) |
| 18-22 | 18.1 (11.7 – 28.0) | 20.0 (12.8 – 31.3) | 21.3 (14.4 – 31.5) | 21.4 (14.3 – 31.9) |
| 23-28 | 20.0 (13.2 – 30.3) | 22.3 (14.6 – 34.1) | 20.0 (13.2 – 30.3) | 20.9 (13.6 – 32.1) |

Prevalence of mood and anxiety disorders in the young people before and after IPW was applied are shown. IPW was conducted to account for any potential bias arising from missing data, so weighted results are presented throughout the main text. Estimated prevalence of disorders were slightly increased when weights were applied, suggesting that unweighted disorder prevalence estimates may be slightly conservative. n=177 at 9-11 years; n=414 at 12-14 years; n=275 at 15-17 years; n=94 at 18-22 years; n=90 at 23-28 years. *CI* confidence interval, *IPW* inverse probability weighting

**A**

**B**

**Figure S2. Rates of MDD and GAD by age group.** The 3-month prevalence of MDD (A) and GAD (B) with IPW applied are shown. The error bars show the 95% confidence intervals. n=177 at 9-11 years; n=414 at 12-14 years; n=275 at 15-17 years; n=94 at 18-22 years; n=90 at 23-28 years. *MDD* major depressive disorder, *GAD* generalised anxiety disorder, *IPW* inverse probability weighting

**Table S5. Prevalence of mood and anxiety disorder by assessment wave**

|  | **Prevalence (%)** | | | |
| --- | --- | --- | --- | --- |
|  | **Wave 1**  **(n 337; mean age=12 years)** | **Wave 2**  **(n 289; mean age=14 years)** | **Wave 3**  **(n 285; mean age=15 years)** | **Wave 4**  **(n 176 for mood; n 144 for anxiety; mean age=23 years)** |
| **Mood disorder** |  |  |  |  |
| Overall | 5.7 | 11.4 | 12.1 | 20.7 |
| Female | 8.0 | 15.0 | 14.8 | 18.4 |
| Male | 2.7 | 6.1 | 8.5 | 24.1 |
| **Anxiety disorder** |  |  |  |  |
| Overall | 14.6 | 15.1 | 15.1 | 25.9 |
| Female | 16.2 | 14.0 | 18.0 | 26.7 |
| Male | 12.2 | 16.7 | 11.3 | 24.3 |

Prevalence of mood disorders and anxiety disorders with IPW applied overall, in females only and in males only for each assessment wave are shown. *IPW* inverse probability weighting

**Figure S3. Mean age at first diagnosis of mood and anxiety disorders.** Mean age at first diagnosis with IPW applied and the range are shown for mood and anxiety disorders. *MDD* major depressive disorder, *NOS* not otherwise specified, *GAD* generalised anxiety disorder, *OCD* obsessive compulsive disorder, *IPW* inverse probability weighting

**Figure S4. The prevalence of disorder comorbidity.** The cumulative prevalence of disorder comorbidity (concurrent disorder) across the four assessment waves of the study with IPW applied is shown for different comorbidity types. ‘Other’ includes eating disorders and personality disorders (schizotypal and borderline). *ADHD* attention deficit hyperactivity disorder*, IPW* inverse probability weighting

**Figure S5. The prevalence of mood disorder by age group when young person report only is used at the adult assessment wave.**  The 3-month prevalence of mood disorder by age with IPW applied when the adult assessment wave (assessment wave 4; mean age 23; age range 18-28) prevalence is estimated using young person report only is shown (solid line). The error bars show the 95% confidence intervals for these estimates. The pattern observed remains similar to the original results (dashed line), where young person and parent reports were combined using an either/or approach. Compared to combined report prevalence, young person report only prevalence of mood disorder is 2% lower at ages 18-22 and 3.8% lower at ages 23-28. n=177 at 9-11 years; n=414 at 12-14 years; n=275 at 15-17 years; n=94 at 18-22 years; n=90 at 23-28 years. *IPW* inverse probability weighting

**Table S6. Rates of reported depressive episodes in between assessment waves**

|  | **Wave 1**  **(n 337)** | **Wave 2**  **(n 289)** | **Wave 3**  **(n 285)** | **Wave 4**  **(n 176)** | **Cumulative (n 337)** |
| --- | --- | --- | --- | --- | --- |
| Rate of MDD at interview | **3.6%**  n=12 | **5.9%**  n=17 | **5.6%**  n=16 | **15.9%**  n=28 | **17.8%**  n=60 |
| Rate of reports of MDD episode before/in between waves | **5.9%**  n=20 | **4.5%**  n=13 | **3.9%**  n=11 | **15.3%**  n=27 | **16.6%**  n=56 |
| Rate of reports of MDD before/in between wave **with no** MDD diagnosis at interview | **5.0%**  n=17 | **2.1%**  n=6 | **3.2%**  n=9 | **13.6%**  n=24 | **9.8%**  n=33 |

The number of young people meeting diagnostic criteria for MDD at each assessment wave interview, the number of those reporting an episode of MDD before the first wave or since the last wave, and the number of those with episodes in between waves who did not meet MDD criteria at interview are shown. For MDD at interview, parent and self-reports were combined using an either/or approach. Only parents reported on the young person’s episodes of MDD occurring in between assessment waves. *MDD* major depressive disorder

**Table S7. Testing for age by assessment wave interaction effects on mood and anxiety disorders**

| **Outcome** | **Age by assessment wave interaction association with outcome**  **(OR, 95% CI, p-value)** |
| --- | --- |
| Mood disorder at any assessment wave | 0.95, 0.89 – 1.01, 0.070 |
| Anxiety disorder at any assessment wave | 1.02, 0.98 – 1.07, 0.400 |

The interaction of young person age and assessment wave was tested as a predictor of mood and anxiety disorders, adjusting for young person age and for assessment wave. No evidence was found of an interaction effect, supporting our use of an approach that assumes no interaction between assessment wave effects and developmental change. *OR* odds ratio, *CI* confidence interval
